# Supplementary material for: Protein Expression of AEBP1, MCM4, and FABP4 Differentiate Osteogenic, Adipogenic, and Mesenchymal Stromal Stem Cells
Source: Int J Mol Sci. 2022 Feb 25;23(5):2568. doi: 10.3390/ijms23052568 (PMC8910760; doi:10.3390/ijms23052568)
Supplement: Supplementary file 1 [file ijms-23-02568-s001.zip › Supplemental_Figures_Sauer_Protein Profiling of MSCs.pdf]

Protein expression of AEBP1, MCM4 and FABP4 differentiate osteogenic, adipogenic and mesenchymal stromal stem cells

Supplemental Figures

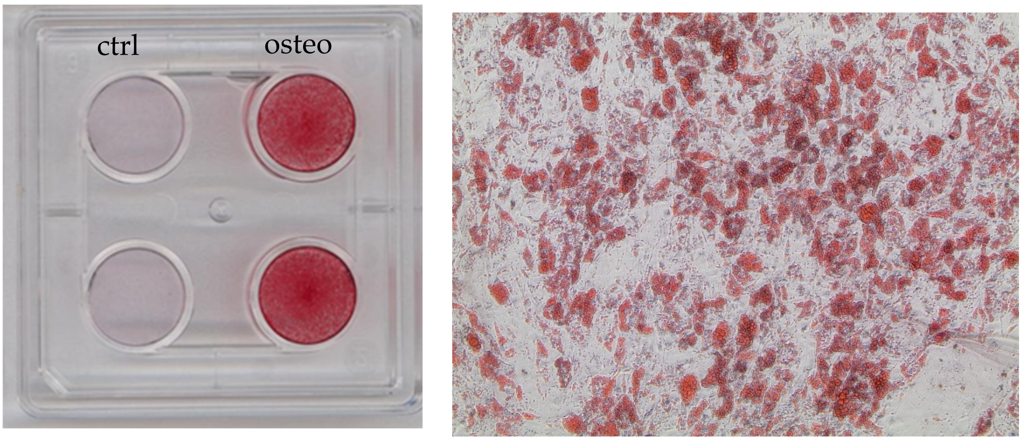

(a) (b)

Supplemental Figure S1. Representative results of functional cell culture stainings. (**a**) Alizarin Red staining visualizing the matrix mineralization of cells (patient p11) after 14 days of osteoblastic differentiation (osteo) and cells incubated with control media (ctrl). (**b**) Oil Red O staining visualizing the lipid droplet area of cells (patient p15) after 14 days of adipocytic differentiation at 10x magnification.

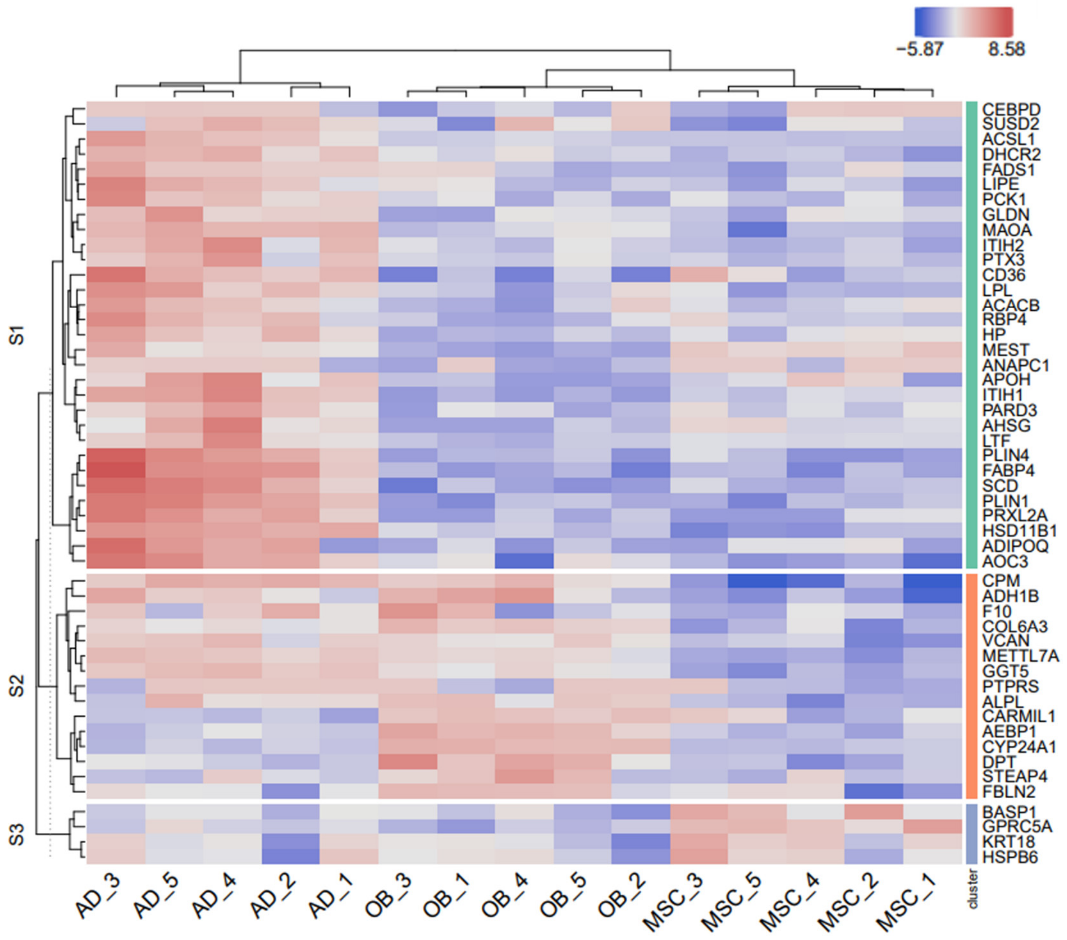

Supplemental Figure S2. Heatmap showing expression of the 50 top differential features (proteins) sorted by two-hierarchical clustering. Red corresponds to relative (feature-wise z-transformed) overexpression, blue to underexpression of the corresponding protein. MSCs, adipocytes and osteoblasts showed substantial differences in their protein expression profiles indicated by the differential clustering behavior into separate populations (x-axis). The dendrogram on the y-axis indicated three protein clusters according to the k-means settings applied (cluster S1-3).

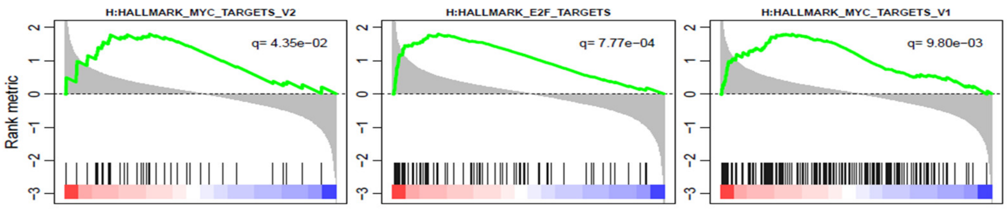

(a)

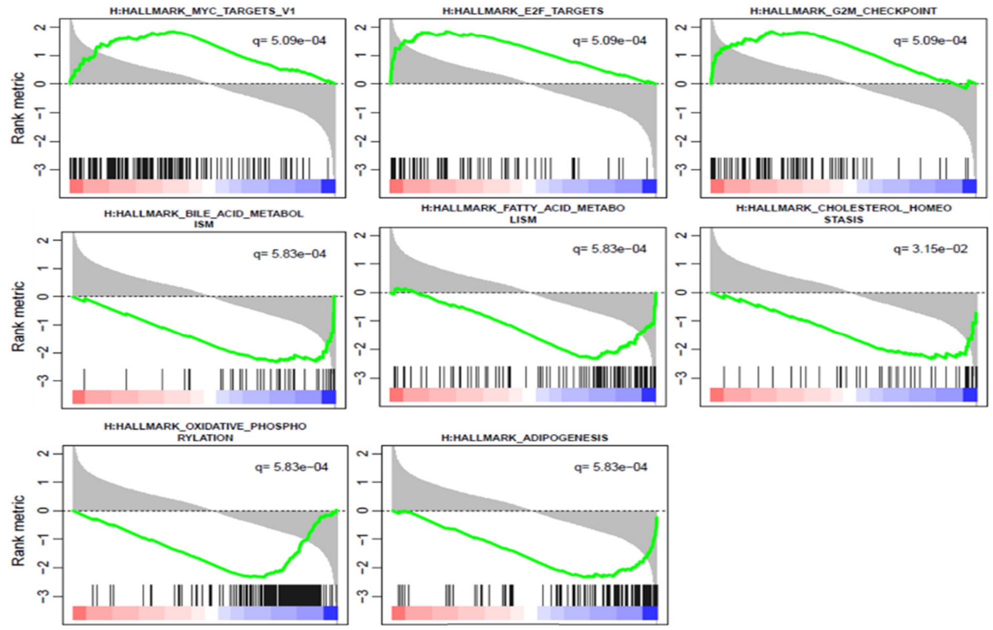

(b)

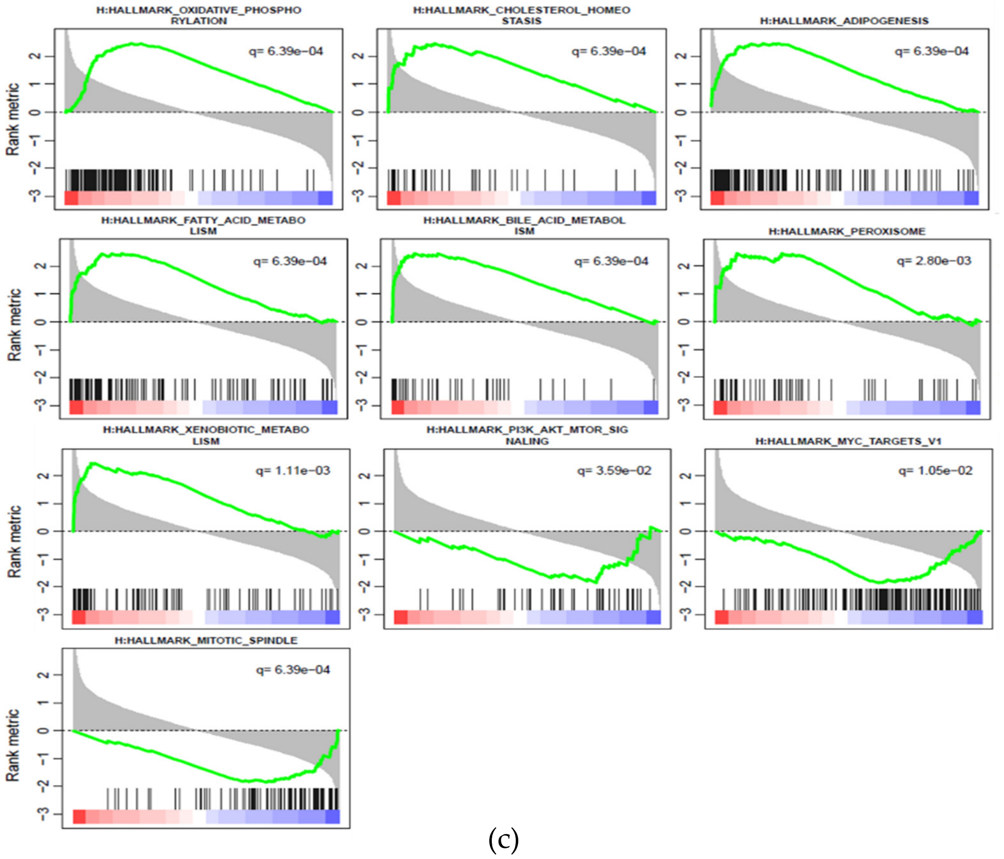

(c)

Supplemental Figure S3. Enrichment plots derived from gene set enrichment analysis (GSEA). 4,108 proteins were searched against the MSigDB hallmark gene set collection. Three genesets were detected as significantly enriched OB/MSC (**a**), nine for the OB/AD (**b**) and ten for the AD/MSC (**c**) comparison (FDR <0.05, $\log_2$FC |>0.2|).

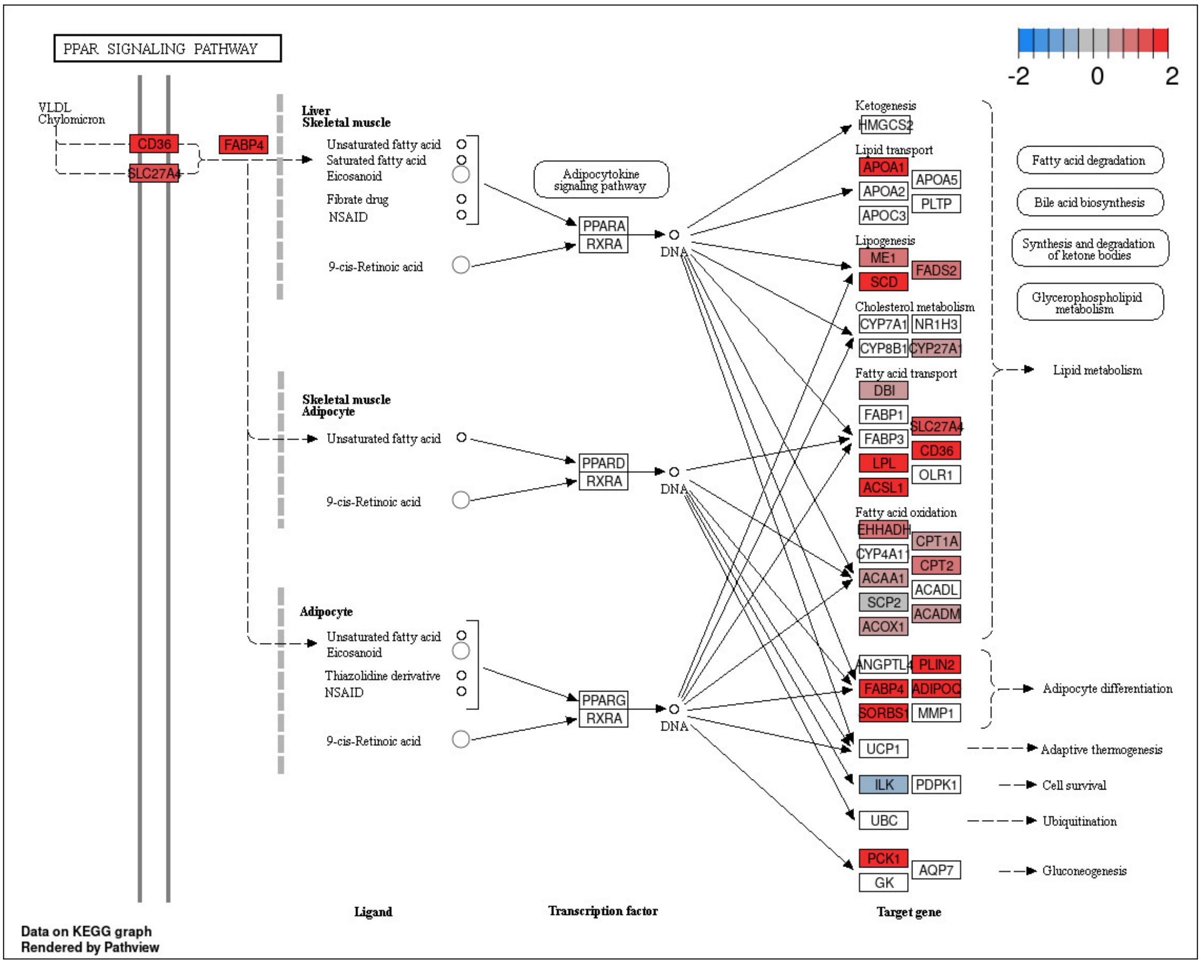

Supplemental Figure S4. Visualization of the PPAR signaling pathway with expression visualization derived from the AD vs. OB comparison with relative, zero-centered log_2FC.
